# Spatiotemporal Evolution of Stress Field during Direct Laser Deposition of Multilayer Thin Wall of Ti-6Al-4V

**DOI:** 10.3390/ma15010263

**Published:** 2021-12-30

**Authors:** Sergei Ivanov, Antoni Artinov, Evgenii Zemlyakov, Ivan Karpov, Sergei Rylov, Vaycheslav Em

**Affiliations:** 1 World-Class Research Center “Advanced Digital Technologies”, St. Petersburg State Marine Technical University, Lotsmanskaya 3, 190121 St. Petersburg, Russia; e.zemlyakov@ltc.ru; 2 Bundesanstalt für Materialforschung und -Prüfung (BAM), Unter den Eichen 87, 12205 Berlin, Germany; Antoni.Artinov@bam.de; 3 National Research Center “Kurchatov Institute”, Akademika Kurchatova 1, 123182 Moscow, Russia; idkarpov@ya.ru (I.K.); rylovsergey25@gmail.com (S.R.); vtem9@mail.ru (V.E.)

**Keywords:** direct laser deposition, finite element simulation, neutron diffraction, residual stresses, Ti-6Al-4V, mechanical properties

## Abstract

The present work seeks to extend the level of understanding of the stress field evolution during direct laser deposition (DLD) of a 3.2 mm thick multilayer wall of Ti-6Al-4V alloy by theoretical and experimental studies. The process conditions were close to the conditions used to produce large-sized structures by the DLD method, resulting in specimens having the same thermal history. A simulation procedure based on the implicit finite element method was developed for the theoretical study of the stress field evolution. The accuracy of the simulation was significantly improved by using experimentally obtained temperature-dependent mechanical properties of the DLD-processed Ti-6Al-4V alloy. The residual stress field in the buildup was experimentally measured by neutron diffraction. The stress-free lattice parameter, which is decisive for the measured stresses, was determined using both a plane stress approach and a force-momentum balance. The influence of the inhomogeneity of the residual stress field on the accuracy of the experimental measurement and the validation of the simulation procedure are analyzed and discussed. Based on the numerical results it was found that the non-uniformity of the through-thickness stress distribution reaches a maximum in the central cross-section, while at the buildup ends the stresses are distributed almost uniformly. The components of the principal stresses are tensile at the buildup ends near the substrate. Furthermore, the calculated equivalent plastic strain reaches 5.9% near the buildup end, where the deposited layers are completed, while the plastic strain is practically equal to the experimentally measured ductility of the DLD-processed alloy, which is 6.2%. The experimentally measured residual stresses obtained by the force-momentum balance and the plane stress approach differ slightly from each other.

## 1. Introduction

The problem of stresses and distortion emerged almost simultaneously with the practical application of additive manufacturing (AM) technologies for the fabrication of parts of any shape and size [1,2,3]. In the last decade, the rapid evolvement of AM technologies in the industry increased the need for more fundamental research on the topic [4,5,6,7,8,9]. Numerous experimental and theoretical studies on the origin of stresses and distortion in AM parts, as well as on the assessment of their effect on the final service properties, have been published in recent years. Despite the considerable successes achieved, many tasks remain challenging but at the same time poorly investigated. In many of them, the primary focus should be on the quantitative estimation of the kinetics of elastoplastic strain and the effects of process parameters and geometry of the manufactured large-size parts on warping and fracture. Existing experimental methods, however, cannot provide comprehensive data due to the limited accessibility for sensors mounting in the regions exposed to high temperatures [10,11,12,13,14,15].

The type I macrostress fields induced by the DLD process are successfully measured by the destructive methods based on stress relaxation effects such as the hole drilling method, the section method (contour method), and the layer removal method [16,17]. Non-destructive techniques such as X-ray diffraction and ultrasonic can only be used to determine stresses in surface layers of the specimens [18,19]. Furthermore, their considerable sensitivity to the microstructural state, or more precisely to the texture, the grain size, and the work hardening, requires precise calibration to ensure a high measurement accuracy. Neutron diffraction is a unique non-destructive method allowing to measure the stress field in the entire sample volume [17,20,21,22,23]. Nevertheless, one main disadvantage of this technique, hindering its application in the industry, is the very high equipment cost. In practice, the experimentally obtained data are often used to validate simulation models developed to predict the stress field evolution in parts of any size and shape.

Initially, there was no particular lack of theoretical background for the mathematical formulation of the problem for stresses and distortion in AM due to its similarity to the well-developed fusion welding processes [24,25]. The challenge, however, was the development of a fast and accurate simulation procedure accounting for specific features typical for the AM process, such as the repeated periodic acting of a highly concentrated moving heat source producing a rather complex spatiotemporal temperature field in the manufactured part. Moreover, the temperature varies from the ambient temperature to the melting point of the deposited material. Such a wide temperature range means also a significant change in the thermo-physical and thermo-mechanical material properties of the buildup.

Improving the accuracy of the simulation models for predicting the stresses and the distortion of AM parts is an important problem. In general, the simulation accuracy is affected by the following factors: (1) assumptions of the mathematical model; (2) finite element discretization of the computational domain; (3) accuracy of the initial and boundary conditions; (4) temperature dependences of the material properties. It is known that the microstructural state and the mechanical properties of DLD-processed alloys differ significantly from the well-studied wrought alloys [26]. Moreover, these depend strongly on the heat input and the inter-pass temperature [27,28]. Thus, the heat accumulation in the buildup is controlled by the deposition strategy and the dwell time. It is shown in [29] that the electron beam melting (EBM)-processed Ti-6Al-4V alloy has lower flow stress than the wrought alloy in the temperature range between 1000 and 1200 °C due to the larger prior β-grain size and thickness of the α-plates. It was found in [30] that the presence of a residual β-phase during the tempering of martensite at elevated temperatures may be the reason for the reduced flow stress of a selective laser melting (SLM)-processed and direct energy deposition (DED)-processed Ti-6Al-4V alloy compared to conventional wrought material with a lamellar microstructure. Furthermore, additively manufactured materials have a faster rate of lamellar-to-globular transformation compared to conventional material. The temperature sensitivity of mechanical properties of a SLM-processed Ti-6Al-4V alloy in a tension test for the temperature ranging from 20 to 550 °C was studied in [31]. Thereby, the ultimate tensile strength of SLM-processed alloy below 500 °C is about 100 MPa higher than that of solution treated and aged alloy and 300 MPa higher than that of annealed alloy. The sensitivity analysis of the thermomechanical response of DLD buildup, presented in [32], revealed that the temperature-dependent material properties play a dominant part in the accuracy of predicted residual stresses and distortion.

There are many theoretical and experimental studies of stresses of DED buildups. However, in the literature the effects of various factors only on the residual stress field are studied. As a rule, there is a significant difference between the process parameters used in scientific studies and those used in the production of large parts in the industry. Lu et al. analyzed numerically the influence of the process parameters on the evolution of longitudinal stresses during DLD [33]. The absence of dwell time between the deposition of the layers resulted in a gradual accumulation of heat in the buildup and the substrate. Thus, as the temperature field was homogenous, the longitudinal stresses in the entire buildup did not exceed 50 MPa in absolute value. Thereby, the highest transient and residual longitudinal stresses corresponded to the upper-side of the base plate near the ends of the buildup. Denlinger and Michaleris analyzed the influence of the dwell time on the residual longitudinal stresses in the middle cross-section of a DLD-processed buildup wall made from the Ti-6Al-4V alloy numerically as well as experimentally [34]. The utilized temperature-dependent mechanical properties with atypically overestimated values of the yield stress and Young’s modulus at temperatures above 700 °C resulted in unrealistically high values of the predicted residual stresses. This problem was overcome by introducing the so-called transformational strain into the model, which negates the stress components at temperatures above 690 °C. Since the considered dwell time of 40 s was not sufficient for cooling the buildup, the obtained residual longitudinal stresses did not exceed 100 MPa. Mukherjee et al. performed a detailed theoretical analysis of stress formation during DLD of a wall consisting of 10 layers using Ti-6Al-4V and IN718 alloys [35]. The verification of the model showed fair agreement with the measured residual stresses. Thereby, the longitudinal residual stress exhibits a steep gradient at both ends of the deposit, making the parts susceptible to buckling and warping. Furthermore, the through-thickness stress, which is responsible for the possible delamination of the component, changes sharply at the substrate deposit interface, while the residual stress changes from tensile to compressive at the layer interfaces. However, these studies lack an in-depth systematic analysis of the stress field evolution during the DLD process.

The present work seeks to extend the level of understanding of the stress field evolution during DLD of a 3.2 mm thick multilayer wall made of Ti-6Al-4V alloy by theoretical and experimental studies. The process conditions were close to the conditions used to produce large-sized structures by the DLD method, resulting in specimens having the same thermal history. A simulation procedure based on the implicit finite element method was developed for the theoretical study of the stress field evolution. The accuracy of the simulation was significantly improved by using experimentally obtained temperature-dependent mechanical properties of the DLD-processed Ti-6Al-4V alloy. The residual stress field in the buildup was experimentally measured by neutron diffraction. The stress-free lattice parameter, which is decisive for the measured stresses, was determined using both a plane stress approach and a force-momentum balance. The influence of the inhomogeneity of the residual stress field on the accuracy of the experimental measurement and the validation of the simulation procedure are analyzed and discussed.

## 2. Materials and Methods

### 2.1. Specimens

The multi-layer wall is a typical buildup geometry used to optimize the process parameters and/or to obtain samples for mechanical testing. Consequently, the buildup size is selected according to the objective of the research. Besides the process parameters, two other parameters, namely the substrate stiffness and the value of the inter-pass temperature, must be selected correctly to obtain manufacturing conditions for the deposition of the multi-layer wall, which are close to those during the manufacturing of a large-sized part.

In DLD of large-size parts, usually, the substrate has a higher thickness and is made from a high-strength alloy, resulting in a high stiffness. Thus, if the DLD-processed alloy has a low ductility, the failure in the part will occur in the regions of stress and strain concentration near the substrate. Therefore, a flexible substrate is preferable in this case. The gradual distortion of the substrate resulting in the spatiotemporal displacement of the manufactured part must be accounted for in the motion path of the processing head, which is the main disadvantage of such an approach. An example of a solution to this problem is shown in [36], where the substrate distortion kinetics, predicted by the FE simulation, was considered in the control program for the robot controller. Combining such an approach with the distortion compensation resulted in a stable DLD process, and thus improved accuracy of the final shape of the manufactured part. Therefore, the most typical case of using a rigid substrate will be further discussed in detail below.

The kinetics of the heat accumulation in the buildup and the substrate are governed by the dwell time. The inter-pass temperature can be used to describe this process. The absence of a pause between the passes results in a gradual increase of the inter-pass temperature, a poor formation of the buildup, and in addition, damages the optical system due to the generation of spatters and overheating of the processing head. Experimental studies on the temperature fields have shown that the inter-pass temperature during DLD of large-sized parts does not exceed 80–100 °C. Therefore, the inter-pass temperature was maintained below 100 °C during the deposition of the buildup.

**Figure 1:**
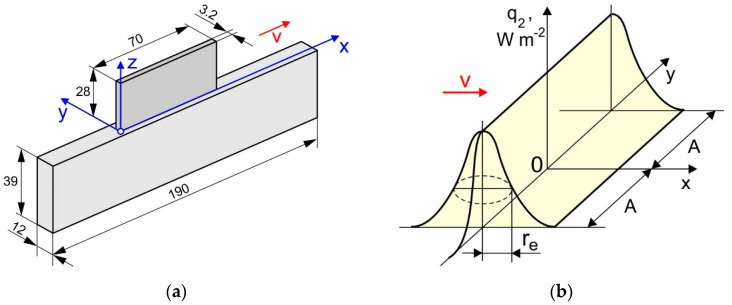
Schematic of (**a**) the sample and (**b**) the power density distribution of a linearly oscillating laser beam.

A Ti-6Al-4V alloy wall was deposited on the edge of a 12 mm thick Ti-6Al-4V plate by the DLD process, see Figure 1a. In total, 50 layers were deposited being 3.2 mm in width, 0.56 mm in height and 70 mm in length, and each of them consisted of a single pass. The final height of the buildup was 28 mm. The in-house robotic DLD machine developed at the St. Petersburg State Marine Technical University in St. Petersburg (Russia) was used for the buildup of the specimens. The machine included a Fanuc 6500 5-axis industrial robot, a rotary table, and a processing head with a discrete coaxial powder feed nozzle and a high-frequency beam oscillation. To prevent oxidation of the specimens during the buildup, the sealed chamber of the machine was filled with argon. Hereby, the residual oxygen content in the chamber did not exceed 100 ppm. The DLD process parameters were as follows: a beam power (*q*) of 2300 W; a beam radius (*r_e_*) of 1.5 mm; amplitude of lateral oscillation of the beam (*A*) of 1.25 mm, see Figure 1b; a process speed (*v*) of 30 mm s^−1^; a powder flow rate of 24 g min^−1^; a gas flow rate of 25 L min^−1^. A unidirectional pass deposition strategy (without changing the deposition direction) was used. The inter-pass temperature was maintained below 100 °C by natural cooling. Note that the inter-pass temperature was measured with K-type thermocouples of 0.5 mm diameter. A plasma rotating electrode processed spherical Ti-6Al-4V powder (particle size: 45–110 μm; average size: 79 μm) was used. The particles have no visible non-metallic inclusions on the surface. The chemical composition was consistent with the standard ASTM F136-02a [37].

### 2.2. Optical and Scanning Electron Microscopy

Optical metallography of etched microsamples was carried out using a Leica DMI8A microscope with a magnification of up to 1000 times. For the etching the Kroll’s reagent (1 mL HF + 2 mL HNO_3_ + 47 mL H_2_O) was used [38]. All metallographic cross-sections were taken from the middle region of the buildup. The Vickers hardness was measured according to the ISO 6507 standard on an FM-310 hardness tester (Future Tech, Kawasaki, Japan) with a load of 3 N. To determine the chemical composition and analyze the microsamples, a Tescan Mira3 scanning electron microscope (TESCAN, Brno, Czech Republic) with an Oxford AZtec console was used (Oxford Instruments NanoAnalysis, Abingdon, UK).

**Figure 2:**
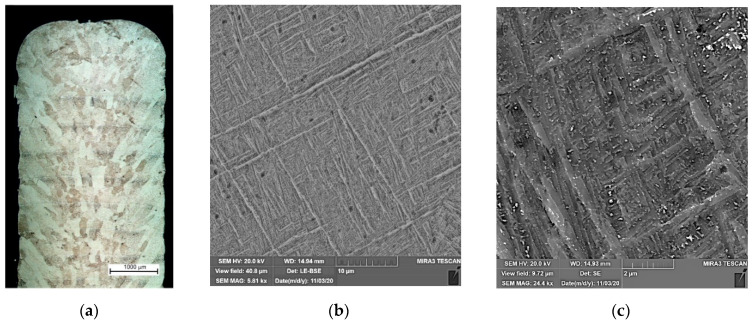
(**a**) Cross-section of the buildup wall; (**b**,**c**) microstructure of the DLD-processed Ti-6Al-4V alloy.

The microstructure of the obtained Ti-6Al-4V buildup is affected by the high crystallization rate of the molten pool due to low inter-pass temperature [27,28] as well as the multiple short-time local reheating from subsequent passes [39,40] and epitaxial crystal growth [41,42]. As can be seen in Figure 2, the microstructure is characterized by thin lamellar α′-phases located perpendicular to each other (martensitic structure). The β-phase cannot be detected in the figure. As was found in [43,44], a small amount of residual β-phase in the form of thin interlayers was revealed in the buildups obtained using similar process parameters. The DLD-processed alloy has increased strength and a decreased ductility due to the presence of an α′-phase. The average microhardness of the buildup alloy was 385 HV0.3.

### 2.3. Tensile Tests at Elevated Temperatures

The mechanical properties of the DLD-processed Ti-6Al-4V alloy at elevated temperatures were obtained using a Gleeble 3800 metallurgical simulation system. The temperature field was controlled by the contact method using a K-type thermocouple of 0.25 mm diameter, discharge spot welded to the surface of the sample. The loading parameters used were a heating rate of 10 °C s^−1^ and a strain rate of 3 mm min^−1^. In addition, an externally mounted sensor was used for a precision recording of the transverse strain with a 500 Hz sampling rate in the central section of the specimen.

**Figure 3:**
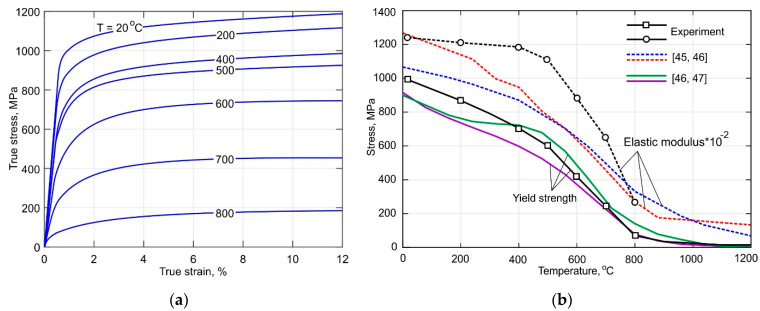
(**a**) True tensile curves of the DLD-processed Ti-6Al-4V alloy; (**b**) comparison of the measured yield strength and Young’s modulus with published data from [45,46,47].

The experimentally obtained true tensile stress curves of the Ti-6Al-4V alloy for the temperature range between 20 and 800 °C are shown in Figure 3a. As seen in Figure 3b, the yield strength decreases drastically by approximately 40% as the temperature increases to 500 °C. The softening rate considerably speeds up during further heating. On the other hand, Young’s modulus remains almost constant at a temperature below 500 °C. However, it decreases drastically by approximately 70% from 109 to 26 GPa as the temperature increases to 800 °C. Comparison of obtained results with published data revealed that the obtained DLD-processed alloy shows significant thermal stability of Young’s modulus than conventional wrought alloy. It is also noteworthy to mention that the properties of the DLD-processed alloy at 800 °C coincide with these of the wrought alloy. Therefore, the data taken from [45,46,47] were used for the temperature above 800 °C.

### 2.4. Thermal Expansion Tests

The temperature-dependent coefficient of thermal expansion (CTE) was obtained using DIL 805 A-D quenching dilatometer test machine. Thereby, a cylindrical specimen with 4 mm diameter and 10 mm length was inductively heated up to 1050 °C at a rate of 3 °C s^−1^ in a vacuum to prevent oxidation. After holding at the peak temperature for 20 min, the specimen was subsequently cooled at a rate of 0.94 °C s^−1^ by blowing it with helium.

**Figure 4:**
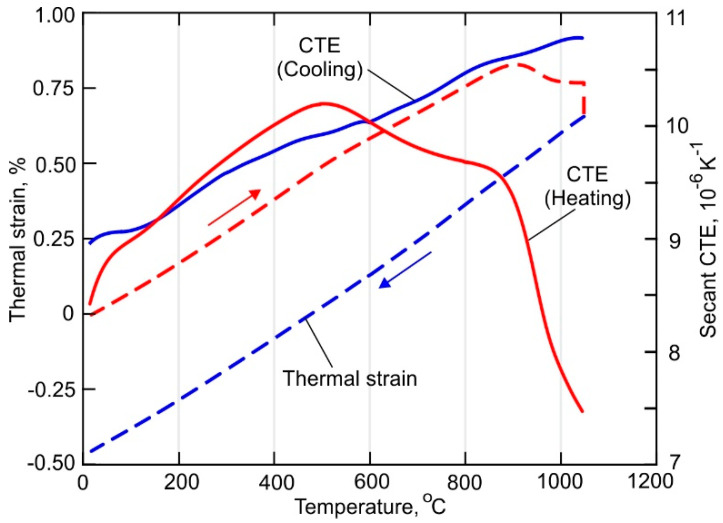
Temperature-dependent thermal strain curve and secant coefficient of thermal expansion of the DLD-processed Ti-6Al-4V alloy.

As can be seen in Figure 4, the heating and cooling parts of experimentally obtained the thermal strain curve have slightly different slopes at a temperature below 600 °C. Further heating causes a decrease of the CTE due to the diffusion-controlled phase transformation α′ → α + β [48]. Note, however, that above 800 °C the α + β → β transformation begins. According to the secant CTE curve, the transformation rate is relatively slow in the temperature range between 800 and 900 °C. At a temperature higher than 900 °C, the rate of β-phase formation sufficiently increases due to an increase in the diffusion mobility of the atoms. During holding at 1050 °C, the β-phase content reaches 100% causes a reduction of the thermal strain. The CTE continuously decreases during the subsequent cooling of the sample. The final microstructure consists of (α + β)-phase formed during the phase transformation β → α + β, which starts at 900 °C and finishes at 810 °C.

### 2.5. Neutron Diffraction Residual Stress Measurements

The neutron diffraction technique is based on measuring the difference between the lattice plane spacing of a stressed material d*_hkl_* and the stress-free material *d*_0,*hkl*_. Direction specific lattice spacing was calculated using Bragg’s law (note that for simplicity, the Miller indices of the crystallographic planes *h*, *k*, and *l* are omitted below):

(1) 2dsinθ=nλ

where *d* is the lattice spacing, *θ* is the diffraction angle, *n* is the integer number, and λ is the incident wavelength of the neutrons.

Since a diffraction peak at a scattering angle 2*θ* corresponds to a lattice spacing *d* the strain can be determined by the difference of the Bragg’s scattering angle in a stressed sample and a stress-free sample [49]:

(2) $$\varepsilon =\frac{d-d_{0}}{d_{0}}=-\left(2\theta -2\theta_{0}\right)\frac{ctg\theta_{0}}{2}$$

The gauge volume is defined by slits in cadmium screens. Usually, the gauge volume contains a large number of crystallites (∼10^7^). Therefore, there is a sufficiently large number of crystallites with the planes (*hkl*) satisfying the conditions of Bragg’s law in the gauge volume of an arbitrarily oriented sample. Since the diffraction peak of the neutrons scattered by the gauge volume is measured, the obtained strain components ε*_x_*, ε*_y_*, ε*_z_* along the three mutually perpendicular principal directions *x*, *y*, *z* are averaged over it. In the next step, the generalized Hooke’s law is used to calculate the stress components σ*_x_*, σ*_y_*, σ*_z_* along these directions as follows:

(3) $$\sigma_{i}=\frac{E}{\left(1+\upsilon \right)\left(1-2\upsilon \right)}\left[\left(1-2\upsilon \right)\varepsilon_{i}+\upsilon \left(\varepsilon_{x}+\varepsilon_{y}+\varepsilon_{z}\right)\right]\;i=x,\;y,\;z$$

where *E* is the Young’s modulus and *ν* is the Poisson’s coefficient. For simplification, the following terms for the stress *σ* and the strain *ε* field components are used in the paper: σ*_x_* and ε*_x_* are the longitudinal components; σ*_y_* and ε*_y_* are the transverse components; σ*_z_* and ε*_z_* are the normal components.

**Figure 5:**
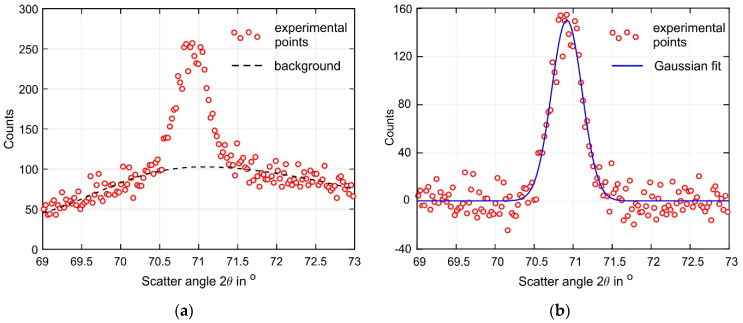
Example of experimental data processing (**a**) before and (**b**) after background removal.

Neutron diffraction measurements were carried out on the STRESS diffractometer at the IR-8 research reactor of the Kurchatov Institute in Moscow (Russia). A double monochromator made of pyrolytic graphite (PG002) and a focusing silicon crystal (Si220) [50,51], which outputs a monochromatic neutron beam with a wavelength λ = 1.548 Å, is used. The diffraction peak α-Ti(103) of titanium at a scattering angle of 2θ ≈ 71.40° was used in the present study, as in lamellar Ti-6Al-4V, the α phase accounts for around 90% of the crystallographic planes at room temperature. The following elastic properties for the α-Ti(103) were used [52,53]: *E*_103_ = 105.5 GPa, *v*_103_ = 0.342. The size and orientation of the gauge volume were chosen to obtain a strain averaged over the thickness of the buildup wall. The gauge volume had the geometrical dimensions of 1.5(*x*) × 3(*y*) × 1.5(*z*) mm^3^. Measurements were carried out at 11 points at a distance *z* = 2 mm from the substrate, corresponding to the following coordinates *x* = (2; 3; 4; 5; 7.5; 10; 12.5; 15; 20; 30; 40) mm. The origin of the coordinate system corresponds to the buildup end at which the depositing layers are initiated, as shown in Figure 1a. The average measurement time for each strain component was approximately 45 min. A non-linear background significantly affecting the accuracy of the diffraction peak position was revealed during data processing. The background fitted by a sixth-degree polynomial was removed and the diffraction peak was approximated by a Gaussian function. The coefficients of the fitting functions in both cases were determined by a non-linear least squares method [54]. An example of the data processing is shown in Figure 5.

Experimentally obtained stresses are strongly affected by the accuracy of the determination of the stress-free lattice parameter. The methods for obtaining the parameter *d*_o_ can be divided into experimental and computational [55]. For experimental determination, a small cube or comb, which is stress-free due to relaxation, is cut out of the studied sample. Since the lattice parameters depend on the local microstructure and chemical composition, it is necessary to cut out and analyze many reference microsamples. In the present work, the interplanar spacing of the stress-free lattice was determined by the following calculation methods based on different assumptions:

***Application of the plane stress approach***

This approach is valid since the thickness of the buildup is small compared to the other dimensions. The interplanar distance for each point of the buildup was determined according to Hooke’s law (3). Considering Expression (2) the stress-free lattice spacing *d*_o_ can be obtained as follows:

(4) $$d_{o}=\frac{1-v}{1+v}d_{z}+\frac{v}{1+v}\left(d_{x}+d_{y}\right)$$

***Application of force-momentum balance***

The force-momentum balance is applied in the selected cross-section. It is valid since external forces are not applied to the studied specimen. For the longitudinal section, where the stresses were measured, this condition can be expressed as:

(5) $$\{\begin{array}{c} \int_{A}\sigma_{z}(x)dA=0\\ \int_{A}^{}x\sigma_{z}(x)dA=0 \end{array},$$

where $\sigma_{z}$ is the normal stress and *A* is the area of the *XY* section of the buildup.

**Figure 6:**
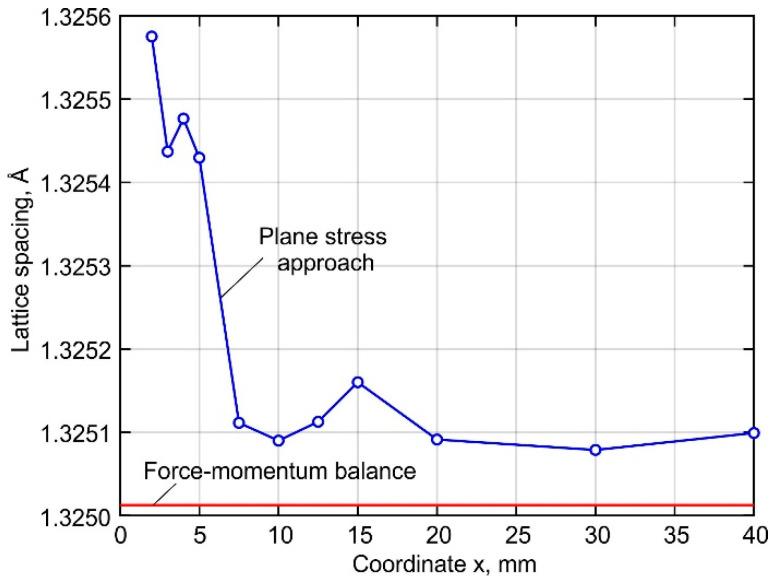
Distribution of stress-free *d_o_* interplanar distance.

The parameter *d_o_* satisfying the system of Equation (5) was obtained by solving an optimization problem, assuming that the stresses at the buildup ends are equal to the yield stress of the material, and the distribution of the normal stress *σ_z_* is symmetrical to the middle cross-section of the buildup. The calculation was performed using the commercial software MATLAB and the Nelder–Mead simplex method [56]. The resulting values of the stress-free interplanar spacing are shown in Figure 6.

### 2.6. DLD Process Modeling

The finite element (FE) simulation of the DLD process is computationally intensive and challenging since a very fine spatial discretization is required to describe the local high-temperature heating due to the highly-concentrated heat/energy source. The resulting computational domain contains hundreds of thousands of degrees of freedom, which requires high computational power. The thermal load created by the depositing passes, which total length can reach several kilometers, must be considered in the calculation of the residual stresses and distortion. Sequentially coupled transient heat conduction and steady elastoplastic problems were solved by an implicit finite element method (FEM) using the commercial FE software Abaqus. The obtained results were processed and visualized using MATLAB. The estimated CPU time for a total of 3.50 m of deposited passes was about 7.14 h for the heat conduction problem and 12.96 h for the elastoplastic problem, using 32Gb RAM Windows PC with an AMD Ryzen Threadripper 1920X 12-core (3.50 GHz) CPU.

**Figure 7:**
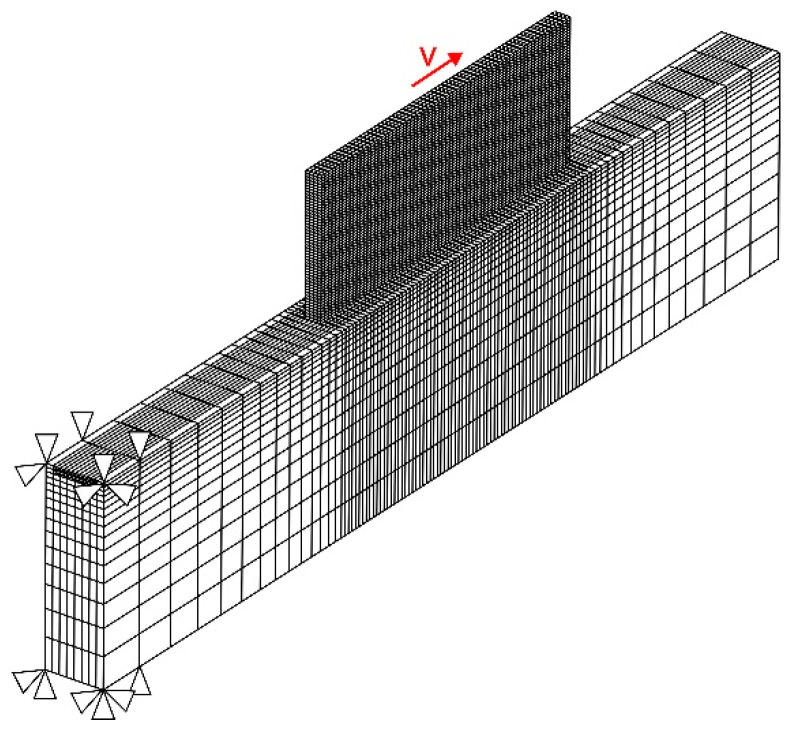
Finite element mesh used for the numerical simulation of DLD process.

The cross-section of each pass was assumed as a fixed rectangle having the average width and height of the experimentally obtained buildup. A computational mesh consisting of 30,072 hexahedral and 8-node elements, resulting in 35,372 nodes in total, is shown in Figure 7. According to the mesh convergence analysis presented in [32,57], the FE mesh has four elements through the pass width and one element through the thickness. Thus, the element size in the buildup area was 1(*x*) × 0.8(*y*) × 0.56(*z*) mm^3^. A coarser mesh was used for the substrate, except for the heat affected zone (HAZ) of the first layer. A uniformly distributed volumetric heat source was moved element-by-element based on the deposition strategy adopted in the experiment. The time step was 0.033 s during the deposition process. However, during the cooling stage between the passes the time step was gradually increased to speed up the calculation procedure. In the simulation of the DLD process, the buildup was gradually deposited at each time step. The “element birth and death” technique was employed in the simulation procedure. The elements belonging to the buildup were deactivated before starting the analysis. At each time step, the activation of the elements was carried out as per to the experimental deposition strategy using a preliminary generated sequence.

The following heat source models are most commonly used to describe the heat input during DLD: (1) Goldak’s volumetric heat source; (2) a uniformly distributed heat source; (3) a concentrated point heat source. The Goldak’s heat source requires a fine mesh accurately accounting for the spatial distribution of the power density. In this case, it is impossible to obtain a solution in a reasonable computational time without using remeshing techniques, even for a small-sized buildup [58,59]. On the other hand, a uniformly distributed heat source provides a reasonably accurate solution even for a coarse FE mesh [32,57]. The concentrated point heat source is the least physically adequate and can lead to convergence difficulties when dealing with steep temperature gradients. Therefore, a uniformly distributed volumetric heat source has been adopted in the present model. The volumetric heat source power density can be expressed as:

(6) $$q_{3}=\frac{\eta P}{V},$$

where *q*_3_ is the volumetric heat source power density, *P* is the heat source power, *η* is the heat source efficiency, assumed to be 0.35 as estimated in [60,61], and *V* is the volume of the heat source.

The unique characteristics of the DLD process make the description of the heat transfer boundary conditions challenging. The heat transfer from the buildup surface is governed by the local action of the concentrated gas-powder jet, as well as by the parameters of the surrounding atmosphere in the working chamber. In its turn, the forced convection parameters of the gas jet are determined by the type of the nozzle, its design features, and the gas flow rate. To the best of the authors knowledge, there are no simulation models and experimental data published, which can be used to describe the influence of all parameters on the heat transfer coefficient. In [62,63], the spatial distribution of the heat transfer coefficient for a coaxial powder feeding nozzle was experimentally established. During the buildup of active alloys, the gradual temperature increase in the chamber filled with argon affects the convective heat transfer. Significant time and resources are required to experimentally determine the influence of the mentioned processes on the heat transfer parameters. Therefore, the unknown value of the heat transfer coefficient was determined by calibration using experimentally measured temperature fields. The adopted value of the heat transfer coefficient was 100 W m^−2^ K^−1^. The boundary at the left end of the substrate was rigidly fixed in the model, as shown in Figure 7.

A multilinear isotropic hardening model without creep effect has been used in the simulation. The temperature-dependent mechanical properties of the DLD-processed Ti-6Al-4V alloy were determined experimentally, except for the Poisson’s ratio, which was taken from [64]. Thereby, the mechanical properties of the substrate, made of the wrought alloy, differed from the buildup only by the Young’s modulus, taken from [35]. On the other hand, the thermo-physical material properties such as heat capacity, thermal conductivity, and mass density were taken the same for the whole model [65,66,67]. The solid-liquid phase transformation was considered in the numerical model by using the latent heat of fusion of 286 J g^−1^ [66].

## 3. Results and Discussion

### 3.1. Longitudinal Distribution of Stresses during the DLD Process

**Figure 8:**
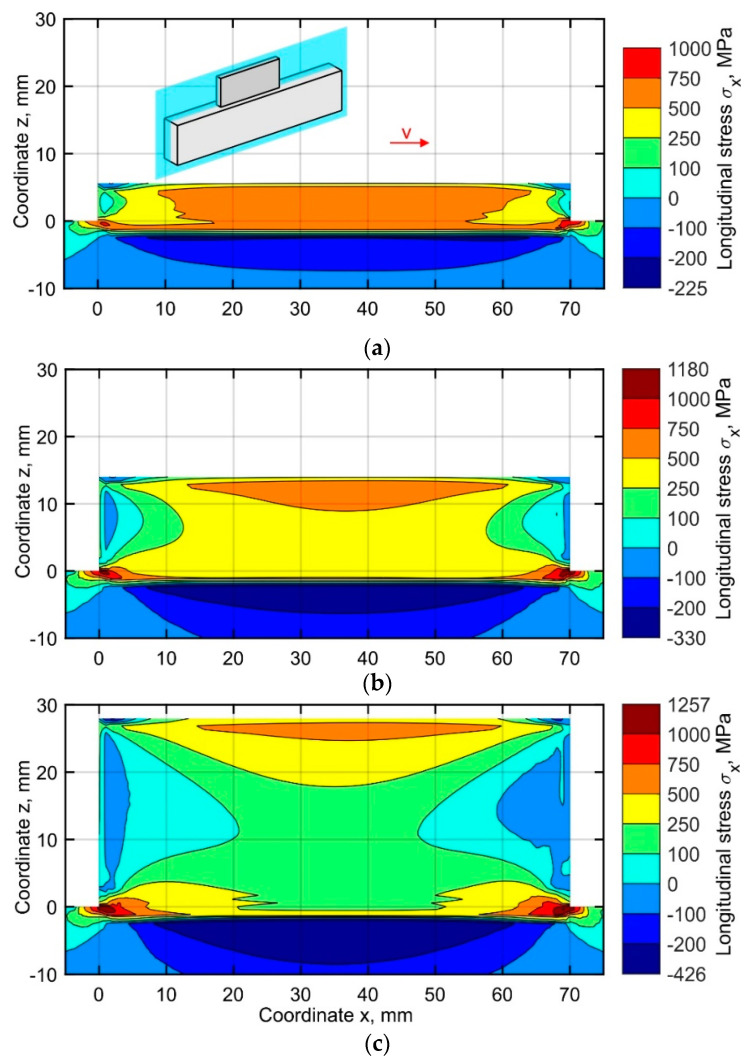
Longitudinal stress distribution in the central longitudinal-section of the buildup at the time corresponding to the end of the cooling after (**a**) 10, (**b**) 25 and (**c**) 50 layers.

The simulated distribution of the longitudinal stresses σ_x_ in the central longitudinal-section of the buildup (located at *y* = 1.6 mm), corresponding to the end of the cooling stage after 10, 25, and 50 layers, are shown in Figure 8a–c. Three regions of tensile stress concentration are observed. One is formed in the recently deposited layer and its HAZ, and the other two correspond to the beginning and the end of the first layer. The mechanism of longitudinal stress formation in the first region is the same for all layers. During the deposition, a narrow area subjected to heating tends to increase its volume due to thermal expansion. Yet, the free expansion of the volume is restricted by the colder metal around it, which leads to the formation of transient compressive stress and plastic strain. During cooling, however, a thermal strain redistribution leads to a thermal contraction of this region. The surrounding cooler metal is strongly resisting the shrinkage of the deposited pass and the corresponding HAZ. Due to the typically low yield stress of metals at high temperatures, the stress will change rapidly from compressive to tensile during the beginning of the cooling stage. During further cooling, the following processes occur: the compressive stresses are replaced by tensile stresses, and the compressive transient plastic strain is partially compensated by the tensile plastic strain. Thus, a tensile stress region is formed along the entire length of the deposited layer and its HAZ. With the increase of the layer number, the longitudinal stiffness is gradually decreasing, resulting in a decrease of the zone size and the peak value of the tensile stress. Therefore, after the deposition of the first ten layers, nearly the entire buildup volume is under tensile stress within the range of 500 to 750 MPa, as shown in Figure 8a. During the further buildup, a gradual narrowing of the area in vertical direction is observed, as seen in Figure 8b,c. The tensile longitudinal stress σ_x_ is balanced by the compressive stress formed in the substrate. The highest tensile longitudinal stress is found in two small regions near the beginning and the end of the first layer. During the buildup process, both the zone size and the peak value of the tensile stress values gradually increase. The residual stress exceeds the yield stress at room temperature by 26%, see Figure 8c, which can be explained by the triaxial stress state, as discussed in the following chapters.

**Figure 9:**
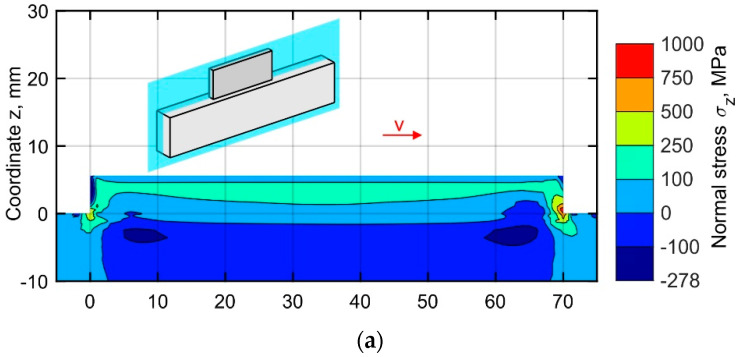

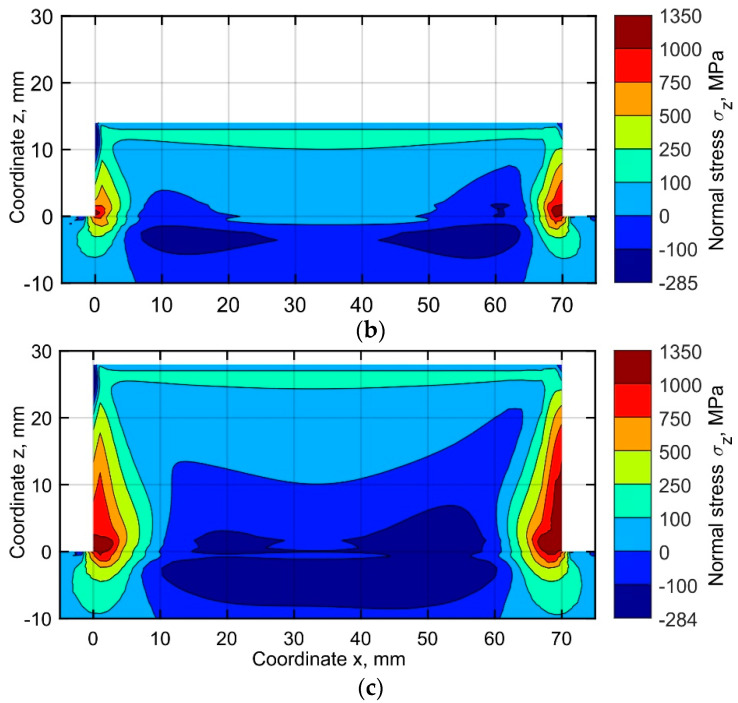
Normal stress distribution in the central longitudinal-section of the buildup at the time corresponding to the end of the cooling after (**a**) 10, (**b**) 25, and (**c**) 50 layers.

The simulated distribution of the normal stresses σ_z_ in the central longitudinal-section of the buildup (located at *y* = 1.6 mm), corresponding to the end of the cooling stage after 10, 25, and 50 layers, are shown in Figure 9a–c. The normal stress has a significantly lower value almost everywhere compared to the longitudinal stress, as the deposited metal expands freely in vertical direction. A distinctive feature is the formation of two regions of tensile stress concentration at the buildup ends. The asymmetry of the field is explained by the unidirectional deposition strategy. As shown in Figure 9, the size of these regions and the peak value of the tensile stress increase layer-by-layer due to the increase of the bending moment generated by the shrinkage of each pass in the longitudinal direction during cooling. A gradual layer-by-layer increase of the bending moment results in an active plastic deformation at the ends of the buildup. The tensile stress is balanced by a region of compressive stresses forming gradually in the substrate and the buildup. The peak tensile residual stresses, exceed the yield stress at room temperature by approximately 35%, corresponding to the end of the buildup on the right-hand side, where the depositing passes are completed.

### 3.2. Through-Thickness Distribution of Stresses during the DLD Process

**Figure 10:**
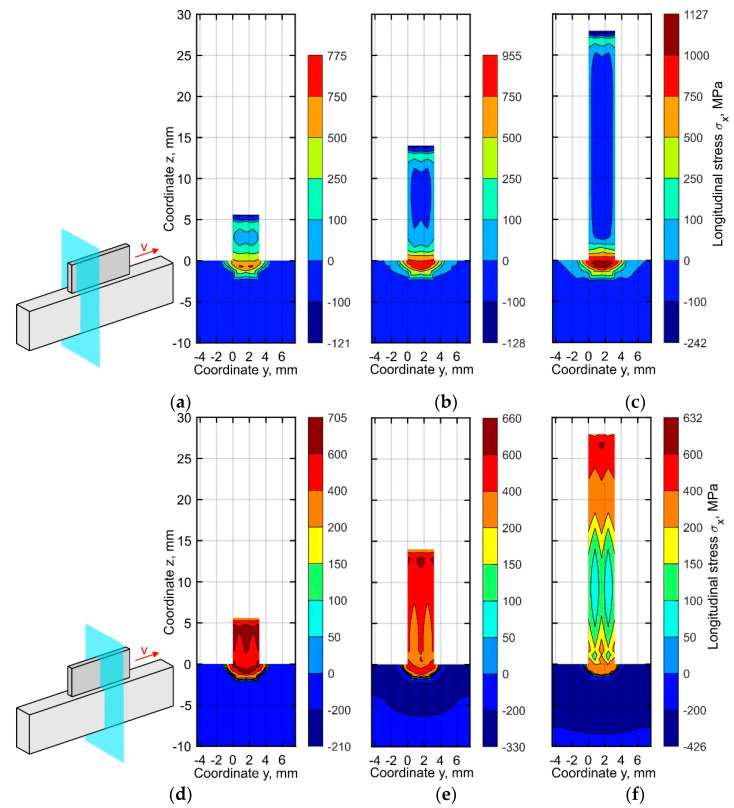
Longitudinal stress distribution in the cross-section (**a**–**c**) near the buildup end, (**d**–**f**) in the middle part at the time corresponding to the end of the cooling after (**a**,**d**) 10, (**b**,**e**) 25, and (**c**,**f**) 50 layers.

**Figure 11:**
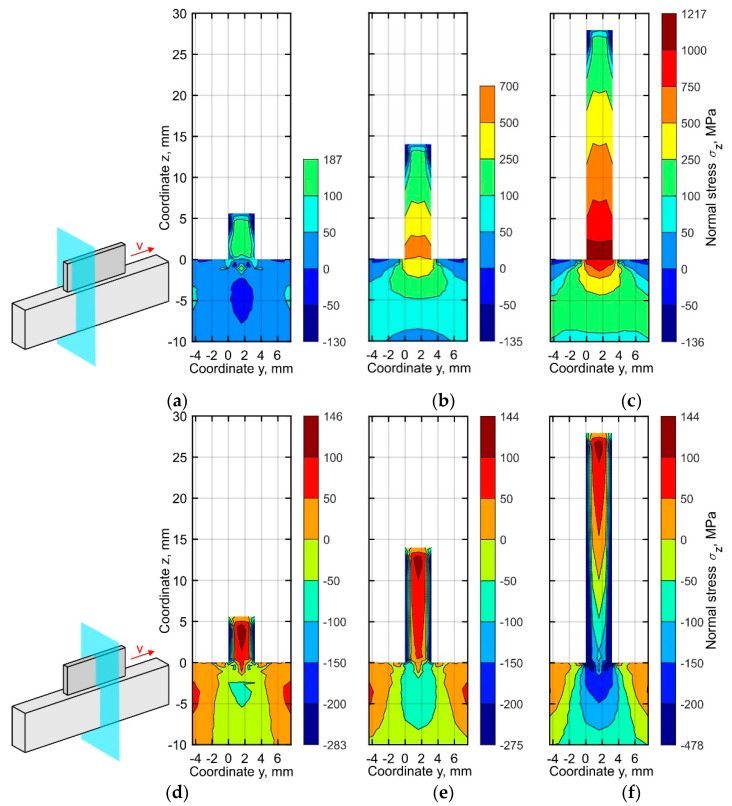
Normal stress distribution in the cross-section (**a**–**c**) near the buildup end and (**d**–**f**) in the middle part at the time corresponding to the end of the cooling after (**a**,**d**) 10, (**b**,**e**) 25, and (**c**,**f**) 50 layers.

**Figure 12:**
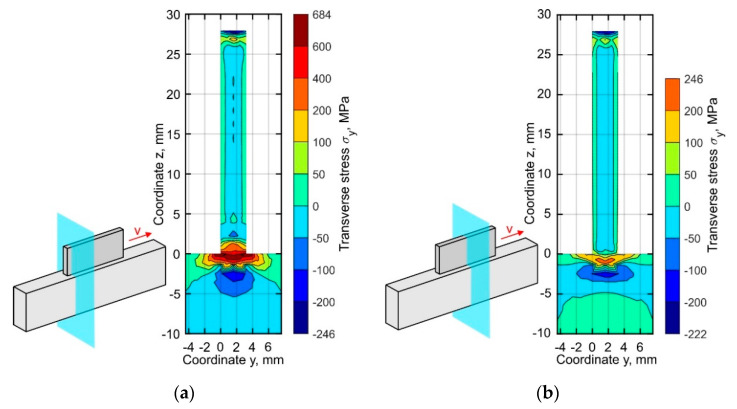
Distribution of the residual transverse stresses σ_y_ in (**a**) the cross-section near the buildup end and (**b**) the middle part of the buildup.

Two cross-sections are considered for the analysis of the distribution of the stress components over the thickness of the buildup during fabrication: (1) a cross-section at *x* = 2 mm near the left end, where depositing passes are beginning and (2) the middle cross-section at *x* = 35 mm, see Figure 10, Figure 11 and Figure 12. The distribution of the longitudinal stress σ*_x_* in the specified sections is shown in Figure 10. After deposition of the first ten layers, the tensile stress is considerably higher in the middle part, see Figure 10d, than near the end of the buildup, see Figure 10a. The stiffness in the longitudinal direction reaches its peak at the middle of the buildup, restricting volume expansion during heating. The non-uniformity of the longitudinal stress distribution over the thickness in the middle section increases during the deposition of the subsequent passes, as seen in Figure 10e,f. However, the stresses are almost uniformly distributed over the thickness near the buildup end, see Figure 10b,c. The region where the longitudinal stress exceeds the yield strength is formed in the substrate and does not involve the metal of the buildup.

The distribution of the normal stresses σ*_z_* through the buildup thickness shows a similar trend. In the middle section, a significant stress non-uniformity is observed as shown in Figure 11d–f. The compressive stress reaches up to 478 MPa near the side surfaces of the buildup. The maximum difference in the stresses along the thickness direction is found near the substrate. Hence, the stress difference between the center and the side surface is 326 MPa at a distance of 2 mm from the substrate, see Figure 11f. On the other hand, a uniform distribution of the normal stress σ_z_ over the thickness of the buildup is observed in the section near the left end (Figure 11a–c), which is similar to the case of the longitudinal stress σ*_x_*.

A detailed analysis of the transverse stresses σ_y_ distribution showed that these vary around 0 MPa by ±50 MPa almost throughout the entire buildup, as shown in Figure 12. The concentration of tensile stress, however, is observed near the top surface of substrate. At the buildup ends, the tensile stress level is found to be several times higher than in the middle section.

**Figure 13:**
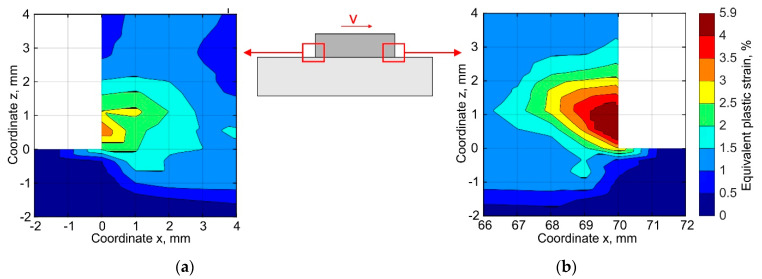
Distribution of the residual equivalent plastic strain near the ends of the buildup corresponding to (**a**) the start and (**b**) end of the pass depositing.

A numerical analysis of the stress evolution has shown that the components of the principal stresses are tensile at the buildup ends near the substrate. Furthermore, the calculated equivalent plastic strain reaches 5.9% near the buildup end, where the deposited layers are completed, see Figure 13, while the plastic strain is practically equal to the experimentally measured ductility of the DLD-processed alloy, which is 6.2%. Thus, in the presence of defects such as pores or lack of fusion, or in the case that the powder is contaminated with impurities reducing its ductility, the buildup will delaminate in the first 2–3 layers.

### 3.3. Validation of Simulation Procedure

**Figure 14:**
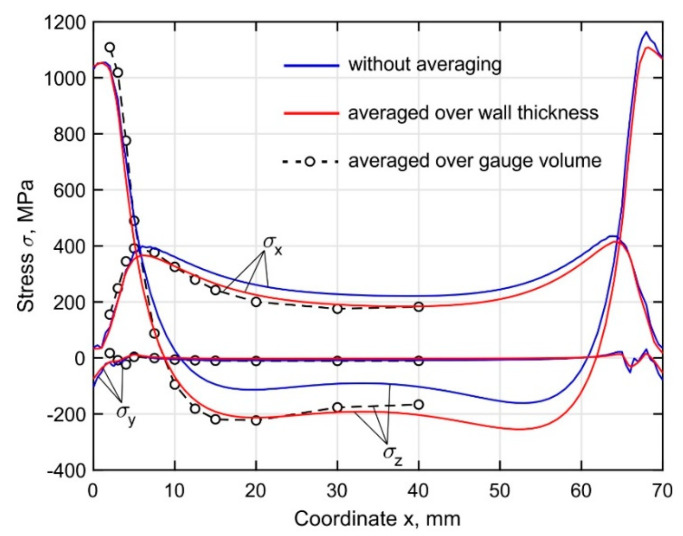
Distribution of the calculated residual stress field components along the length of the buildup at a distance of 2 mm from the substrate.

The experimental methods of stress measurement provide averaged values of the stresses along the gauge length or volume. In the case of the neutron diffraction method, the gauge volume was 1.5(*x*) × 3(*y*) × 1.5(*z*) mm^3^. It is important to understand that the real residual stress field is inhomogeneous and averaged during the experimental measurements, which can have subsequent influence on the validation of the simulation procedure. Figure 14 shows the distribution of the calculated residual stresses along the length of the buildup at a distance of 2 mm from the substrate for the following cases: (1) without averaging, i.e., stress distribution along the line with coordinates *y* = 1.6 mm and *z* = 2 mm; (2) after averaging over the buildup thickness; (3) after averaging according to the gauge volume dimensions at the points of experimental stress measurement. Obviously, the value of the resultant stresses averaged over the wall thickness is affected only by the stress distribution over the thickness, i.e., in the transverse direction along the *y*-axis. In the case of averaging over the gauge volume, an additional effect is produced by the stress gradients in vertical and longitudinal directions. The normal stress σ_z_ curve is not balanced without averaging due to the force-momentum equilibrium condition, which must be satisfied for the entire longitudinal section rather than for a single line. At a distance of more than 10 mm from the buildup ends, a discrepancy between the curves is observed, caused by an increase in the non-uniformity of the through-thickness stress distribution, as observed in Figure 10, Figure 11 and Figure 12. The curves representing the averaged results show a balanced distribution. At a distance of less than 5 mm from the buildup ends, the curves practically merge, as the stresses are uniformly distributed across the thickness.

**Figure 15:**
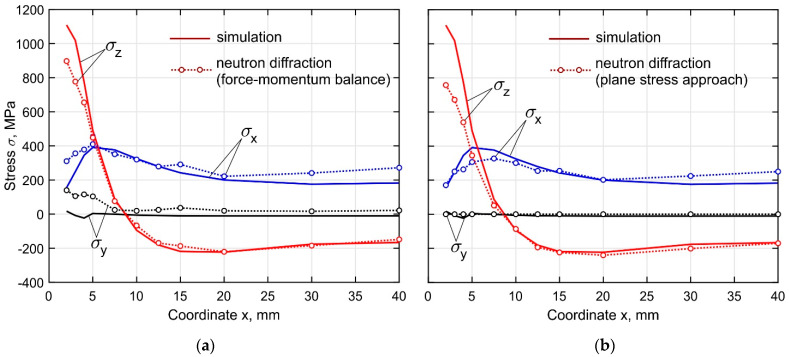
Comparison of the numerically obtained and experimental measured curves of the residual stresses components along the length of the buildup at a distance of 2 mm from the substrate. (**a**)~force-momentum balance approach. (**b**) plane stress approach.

The experimentally measured residual stresses are shown in Figure 15. By the performed strain measurements with the neutron diffraction method, an average accuracy of approximately 5 × 10^−5^ was achieved, providing stress accuracy of 10 MPa in terms of the calculated stresses (errors only due to the neutron counting statistics). Furthermore, the error bar is approximately equal to the diameter of the circles in the figure, and therefore not shown. It is seen that the curves obtained by the force-momentum balance and the plane stress approach differ slightly from each other. The main uncertainties observed near the buildup end are explained by the significant plastic deformations and the triaxial stress state in this region. The residual transverse stress σ_y_ obtained with the force-momentum conditions is 26 MPa over the entire length of the buildup except for the end where it reaches 115 MPa. The experimentally measured residual stress curves were compared with the numerically obtained data, averaged over the gauge volume. The calculated curves of the longitudinal and normal stresses are in good agreement with the experimental curves. The main uncertainties are found near the buildup end. At the point with coordinate *x* = 2.0 mm, the discrepancy of the normal stresses for the cases using the force-momentum and the plane stress approach reaches 200 and 340 MPa, respectively. In the developed simulation procedure of the DLD process, experimentally measured temperature-dependent mechanical properties of the final DLD-processed Ti-6Al-4V alloy are utilized, to increase the accuracy of the predicted stresses and distortion of the buildup.

## 4. Conclusions

The present work seeks to extend the level of understanding of the stress field evolution during DLD of a 3.2 mm thick multilayer wall made of Ti-6Al-4V alloy by theoretical and experimental studies. The process conditions were close to the conditions used to produce large-sized structures by the DLD method, resulting in specimens having the same thermal history. A simulation procedure based on the implicit finite element method was developed for the theoretical study of the stress field evolution. The accuracy of the simulation was significantly improved by using experimentally obtained temperature-dependent mechanical properties of the DLD-processed Ti-6Al-4V alloy. The residual stress field in the buildup was experimentally measured by neutron diffraction. The stress-free lattice parameter, which is decisive for the measured stresses, was determined using both a plane stress approach and a force-momentum balance. The influence of the inhomogeneity of the residual stress field on the accuracy of the experimental measurement and the validation of the simulation procedure are analyzed and discussed. The following conclusions are drawn:

1. The non-uniformity of the through-thickness stress distribution reaches a maximum in the central cross-section, while at the buildup ends, the stresses are distributed almost uniformly.

2. The components of the principal stresses are tensile at the buildup ends near the substrate. Furthermore, the calculated equivalent plastic strain reaches 5.9% near the buildup end, where the deposited layers are completed while the plastic strain is practically equal to the experimentally measured ductility of the DLD-processed alloy, which is 6.2%. Thus, in the presence of defects such as pores or lack of fusion, or in the case that the powder is contaminated with impurities reducing its ductility, the buildup will delaminate in the first 2–3 layers.

3. The experimentally measured residual stresses obtained with the force-momentum balance and the plane stress approach differ slightly from each other. The calculated curves of the longitudinal and normal stresses agree well with the experiments.
